# Building a transgenic sexing strain for genetic control of the Australian sheep blow fly *Lucilia cuprina* using two lethal effectors

**DOI:** 10.1186/s12863-020-00947-y

**Published:** 2020-12-18

**Authors:** Ying Yan, Maxwell J. Scott

**Affiliations:** 1 Department of Entomology and Plant Pathology, North Carolina State University, Campus Box 7613, Raleigh, NC 27695-7613 USA; 2 Department of Insect Biotechnology in Plant Protection, Justus-Liebig-University Giessen, Institute for Insect Biotechnology, Winchesterstraße 2, 35394 Giessen, Germany

**Keywords:** Sterile insect technique (SIT), Tetracycline transactivator (tTA), Head involution defective (hid), Genetic pest management

## Abstract

**Background:**

The sterile insect technique (SIT) has been successfully used in many pest management programs worldwide. Some SIT programs release both sexes due to the lack of genetic sexing strains or efficient sex separation methods but sterile females are ineffective control agents. Transgenic sexing strains (TSS) using the tetracycline-off control system have been developed in a variety of insect pests, from which females die by either of two commonly used lethal effectors: overexpression of the transcription factor *tetracycline transactivator* (tTA) or ectopic expression of a proapoptotic gene, such as *head involution defective* (*hid*). The lethality from tTA overexpression is thought to be due to “transcriptional squelching”, while *hid* causes lethality by induction of apoptosis. This study aims to create and characterize a TSS of *Lucilia cuprina*, which is a major pest of sheep, by combining both lethal effectors in a single transgenic strain.

**Results:**

Here a stable TSS of *L. cuprina* (DH6) that carries two lethal effectors was successfully generated, by crossing FL3#2 which carries a female-specific tTA overexpression cassette, with EF1#12 which carries a tTA-regulated *Lshid*^*Ala2*^ cassette. Females with one copy of the FL3#2 transgene are viable but up to 99.8% of homozygous females die at the pupal stage when raised on diet that lacks tetracycline. Additionally, the female lethality of FL3#2 was partially repressed by supplying tetracycline to the parental generation. With an additional *Lshid* ^*Ala2*^ effector, the female lethality of DH6 is 100% dominant and cannot be repressed by maternal tetracycline. DH6 females die at the late-larval stage. Several fitness parameters important for mass rearing such as hatching rate, adult emergence and sex ratio were comparable to those of the wild type strain.

**Conclusions:**

Compared to the parental FL3#2 strain, the DH6 strain shows stronger female lethality and lethality occurs at an earlier stage of development. The combination of two tTA-dependent lethal effectors could improve strain stability under mass rearing and could reduce the risk of resistance in the field if fertile males are released. Our approach could be easily adapted for other pest species for an efficient, safe and sustainable genetic control program.

**Supplementary Information:**

The online version contains supplementary material available at 10.1186/s12863-020-00947-y.

## Background

Genetic control methods like the sterile insect technique (SIT) have been used worldwide to battle insect pests. Some SIT programs release both sexes but sterile females are ineffective control agents since they compete with wild females for mating with sterile males [1, 2]. Additionally, release of sterile fruit fly females can be problematic as “sterile stings” can lead to damaged fruit as a consequence of microbial growth at the site of puncture [3]. To achieve male-only release, transgenic sexing strains (TSS) have been developed in a variety of agricultural pests and human-disease vectors [4]. The general strategy to build a TSS is to incorporate a female-specific (FS) element and a lethal effector into the binary tetracycline-off (Tet-off) system. The FS element can be a promoter/enhancer [5, 6] or an alternatively spliced intron which is typically derived from the *transformer* (*tra*) sex determination gene [7–9]. In a single-component sexing system, the sex-specific *tra* intron is inserted within the *tetracycline transactivator* (tTA) gene such that only the female splice variant encodes functional tTA protein. Expression is driven by a tetracycline operator (tetO)-core enhancer-promoter sequence, thus forming an auto-regulated system as binding of tTA to tetO enhances tTA expression. Very high levels of tTA are lethal, possibly due to “transcriptional squelching” and/or interference with ubiquitin-dependent proteolysis [7, 10]. In a two-component sexing system, a pro-apoptotic gene such as *head involution defective* (*hid*) is driven by the tetO-core enhancer-promoter (effector). The *tra* intron is inserted within the *hid* gene such that only the female transcript encodes a functional HID protein. A gene promoter that is mostly active in early embryos is used to drive tTA expression (driver). Binding of tTA to tetO activates *hid* expression causing female embryo lethality due to high levels of apoptosis [9, 11, 12]. For both systems, only females die when the tetracycline is absent from the diet. Females are fully viable and fertile if tetracycline is added to the insect diet as the antibiotic inhibits binding of tTA to tetO [7, 9–13]. Consequently, the TSS can be maintained in the SIT factory by supplementing the mass rearing diet with tetracycline.

The Australian sheep blow fly *Lucilia cuprina*, is a major pest of sheep and causes considerable economic loss in Australia and New Zealand [14, 15]. SIT was used to successfully eradicate the New World screwworm *Cochliomyia hominivorax*, a blow fly that is related to *L. cuprina*, from North and Central American over a 50-year program [16]. This was regarded as a significant achievement in insect pest management history [17]. Consequently, genetic control methods were proposed for the control of *L. cuprina* [18]. *L. cuprina* TSSs were initially developed using the tTA overexpression system with sex-specificity achieved using the first intron from the *C. hominivorax transformer* (*Chtra*) gene [19]. Female lethality was at the late larval/pupal stages [20]. More recently, *L. cuprina* transgenic embryonic sexing strains (TESS) were established using the two-component system, in which the promoters from the *L. sericata* cellularization genes *bottleneck* (*Lsbnk*) or *nullo* were used to drive tTA expression and the effector gene *Lshid* was interrupted by *Chtra* intron [12, 21]. Females carrying both driver and effector components died at the embryo stage if given diet that lacked tetracycline. The gene constructs evaluated in *L. cuprina* were also used to make *C. hominivorax* TSS and the most efficient strains are currently being evaluated for potential field application [12, 20, 22, 23].

Although successful in the laboratory at a small scale, the efficacies of the TSS are subjected to genetic mutations that could hinder the function of a lethal effector. For a Tet-off female lethality system, spontaneous mutations were calculated to occur in the effector genes at a 1 in a million frequency [24]. Currently, more than 15 million sterile *C. hominivorax* are released per week along the Panama-Colombia border, to prevent the re-invasion of *C. hominivorax* from South America [17]. Breakdown of the TSS during mass rearing due to genetic mutation could lead to the release of females. This would be particularly problematic if the radiation step is omitted, which would produce some savings for the program [23]. Further, release of fertile males carrying a single dominant female lethal gene is predicted from modeling to be more efficient than SIT [6, 25, 26]. This is mostly because the male offspring of the released males could mate with wild females and pass on the dominant female lethal gene to half of their offspring. However, release of fertile males with a single effector could also fail in the field due to preexisting genetic alleles in the targeted population that provide resistance to the lethal mechanism [27]. The tTA overexpression system is sensitive to the genetic background of the population [23, 28]. Similar concerns apply to the use of insecticides. Indeed, pre-existing alleles associated with resistance to malathion were found in *L.* cuprina [29]. Thus, development of TSS with multiple lethal effectors or redundant lethal systems would be very advantageous for an efficient, safe and sustainable genetic control program [24, 30]. In the present study, two lethal effectors from the single and two-component systems, were combined in a single transgenic strain of *L. cuprina*. Specifically, the aims of this study were to determine if it is possible to breed a stable homozygous strain that carries the two lethal effectors, and if such strain could enhance the lethal effect and kill females at an earlier developmental stage compared to the parental strain with the single component system.

## Results

### A TSS carrying the two-lethal effectors showed dominant female lethality

**Fig. 1:**
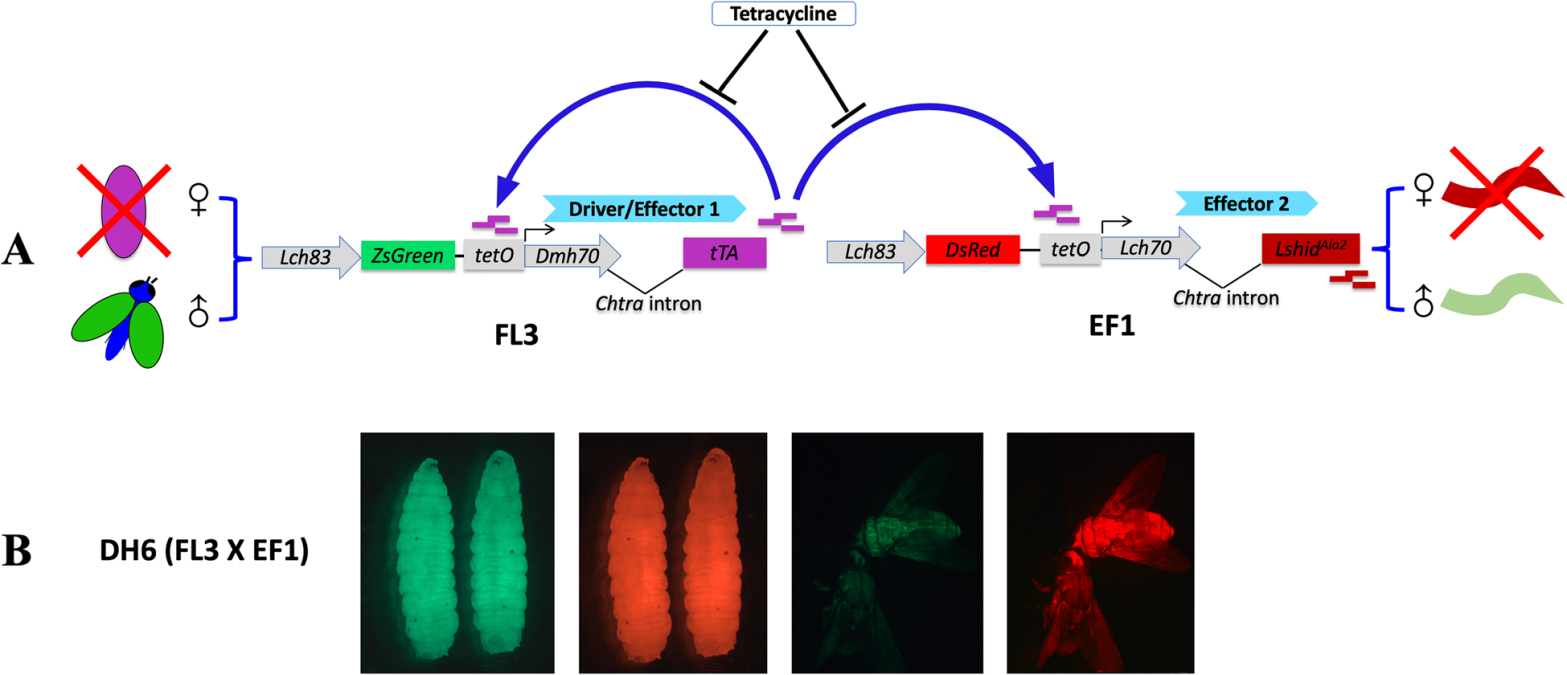
*L. cuprina* transgenic sexing strain DH6 carrying the two lethal effectors. **a** Schematic illustration of the two lethal effectors strategy. The FL3 *piggyBac* construct contains a ZsGreen marker gene driven by *Lchsp83* promoter and a sex-specific tTA overexpression cassette (tetO21-*Dmhsp70* core-*Chtra* intron-tTA-SV40 polyA). The EF1 *piggyBac* construct contains a DsRed marker gene driven by the *Lchsp83* promoter and a sex-specific *Lshid* ^*Ala2*^ effector cassette (tetO21-*Lchsp70* core-*Chtra* intron-*Lshid* ^*Ala2*^-SV40 polyA). In the absence of tetracycline, tTA is overexpressed from the FL3 transgene causing female lethality at the pupal stage. However, in the two lethal effectors strategy tTA would also activate expression of *Lshid* ^*Ala2*^, which acts as the second lethal effector. Consequently, females die at an earlier late-larval stage because of activation of apoptosis. **b** DH6 (FL3#2; EF1#12) shows both green and red fluorescence in third instar larvae and young adults

To build a *L. cuprina* TSS with the two lethal effectors, the female-lethal (FL) strain FL3#2 that carries a sex-specific tTA overexpression cassette [20] and an effector strain EF1#12 that carries a sex-specific *Lshid* ^*Ala2*^ cassette [12], were selected for crossing and breeding (Fig. 1a). A double homozygous (DH) strain DH6 was successfully generated by screening the wandering third instar larvae based on the fluorescence intensity of the ZsGreen and DsRed whole body marker genes (Fig. 1b). DH6 was stably maintained in the lab on diet supplemented with tetracycline (100 μg/mL) for at least 3 years. On tetracycline, the adult emergence ratio (percentage of pupae that develop into adults) was 86.2, which is comparable to the parental FL3#2 line and DH strains developed previously with embryo tTA driver lines (Table 1). Further 48.4% of the adults were female, showing that females are fully viable on diet with tetracycline.

**Table 1:** Rearing efficiency and female lethality of *L. cuprina* TSS

TSS	Condition	Tet^**a**^	#Pupae	#Male	#Female	#Adult	AER^**b**^	%Male
**FL3#2**^**c**^	Heterozygous	–	2098	644	735	1379	65.7	46.7%
		+	2468	1068	1048	2116	85.1	50.5%
	Homozygous	–	2387	1029	1	1030	43.1	99.9%
		+	1647	672	701	1373	83.3	49.0%
**FL3#2**^**d**^	Homozygous	–	1736	881	2	883	50.9	99.8%
		+	1562	629	685	1314	84.1	47.9%
**DH6**^**d**^**(FL3#2; EF1#12)**	Heterozygous	–	1717	1383	0	1383	80.5	100%
	Homozygous	–	427	270	0	270	63.2	100%
		++/−	1421	885	0	885	62.3	100%
		+	1611	719	669	1388	86.2	51.8%
**DH1**^**e**^**(DR2#6; EF1#12)**	Heterozygous	–	1091	916	0	916	84	100%
	Homozygous	+/−	1054	958	0	958	90	100%
		+	2719	1284	1234	2518	92.6	51.0%
**DH2**^**e**^**(DR2#6; EF3E)**	Heterozygous	–	1252	1099	0	1099	87.8	100%
	Homozygous	+/−	694	594	0	594	85.6	100%
		+	1724	784	738	1522	88.3	51.5%
**DH3**^**f**^**(DR3#2; EF3E)**	Heterozygous	–	1082	771	0	771	71.3	100%
	Homozygous	+/−	1158	981	0	981	84.7	100%
		+	1949	838	810	1648	84.6	50.8%
**DH4**^**f**^**(DR3#4; EF1#12)**	Heterozygous	–	2398	1690	30	1720	71.7	98.3%
	Homozygous	+/−	953	801	0	801	84.1	100%
		+	2284	1039	1008	2047	89.6	50.8%
**DH5**^**f**^**(DR5#4; EF1#12)**	Heterozygous	–	1271	1132	0	1132	89.1	100%
	Homozygous	–	660	561	0	561	85	100%
		+	2162	981	881	1862	86.1	52.7%

^a^ “-” stands for no tetracycline in the diet, and “+” stands for plus tetracycline in the diet, “+/−” indicates parents fed a low dose of tetracycline (1 or 3 μg/mL for the first 2 days), “++/−” indicates a high dose of tetracycline (100 μg/mL) was supplied to the parental adults for the first 8 days but not their progeny that were counted

^b^AER stands for adult emergence ratio

^c^Data from [20]. Eggs were collected up to two times from 10 to 20 pairs of adults

^d^Data from this study, three replicates of 8-pairs per cage

^e^Data from [12]. For DR2 the *bottleneck* (*bnk*) cellularization gene promoter from *L. sericata* was used to drive expression of tTA. EF3 contains the wild type version of *Lshid* whereas EF1 has a phosphomutated version called Lshid^Ala2^

^f^Data from [31]. The *spitting image* (*spt*) gene promoter from *L. sericata* and the *actin5C* gene promoter from *L. cuprina* was used to drive expression of tTA for DR3 and DR5, respectively

**Fig. 2:**
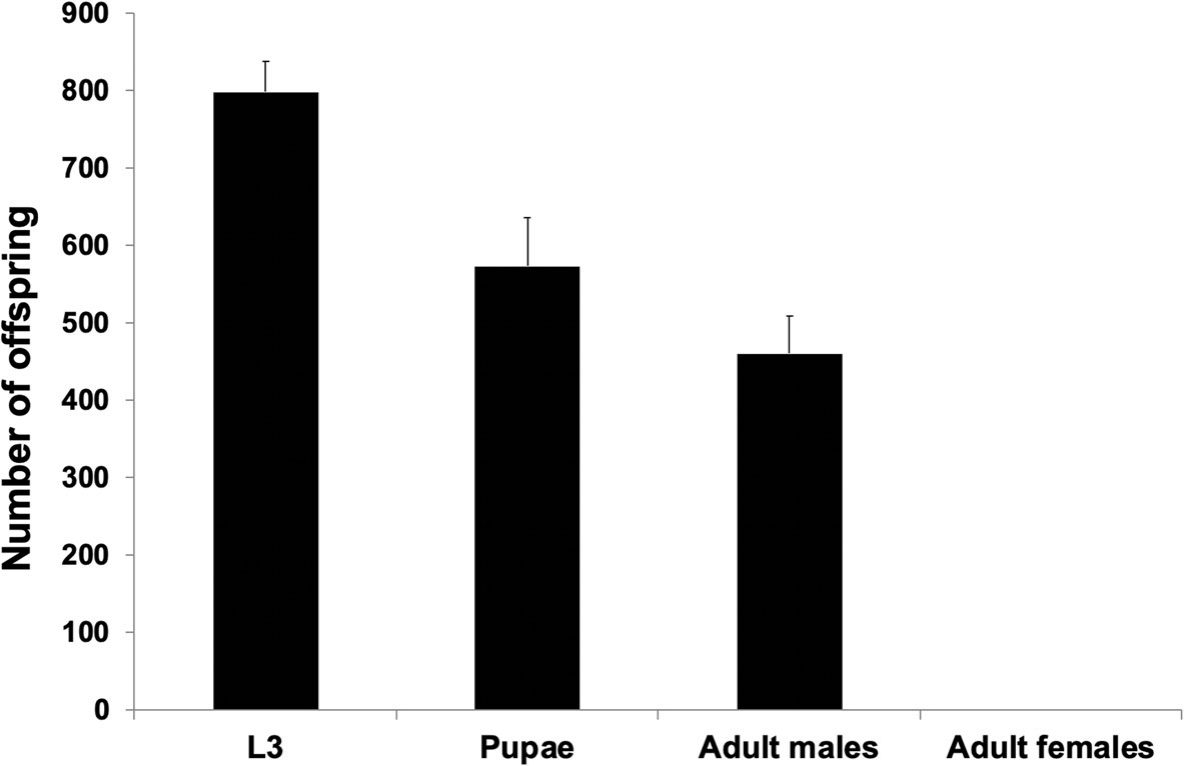
Female-specific lethality of DH6 with one copy of each transgene. Eight homozygous DH6 males were crossed with eight WT virgin females and their offspring raised on diet without tetracycline. The number of wandering third instar larvae (L3), pupae and adult male and female offspring from each cross were counted. Each experiment was performed three times. Mean ± standard deviation are shown

When raised on diet without tetracycline, we previously found that females with one copy of FL3#2 were viable but 99.9% of females with two copies of the transgene died at the pupal stage [20]. After several years in culture, we tested FL3#2 again for female lethality and similar results were obtained (Table 1), which suggested that the killing efficiency of tTA overexpression is stable in this line. When raised in the absence of tetracycline, 100% of heterozygous DH6 females with one copy of each transgene died (Fig. 2, Table 1). Thus, the lethal effect was largely enhanced when compared to that of FL3#2 (Table 1). Additionally, it appears that heterozygous females died at a larval stage as most pupae emerged into males (84.0%, Table 1).

### The female lethality of the TSS carrying the two lethal effectors cannot be inhibited by maternal tetracycline

**Fig. 3:**
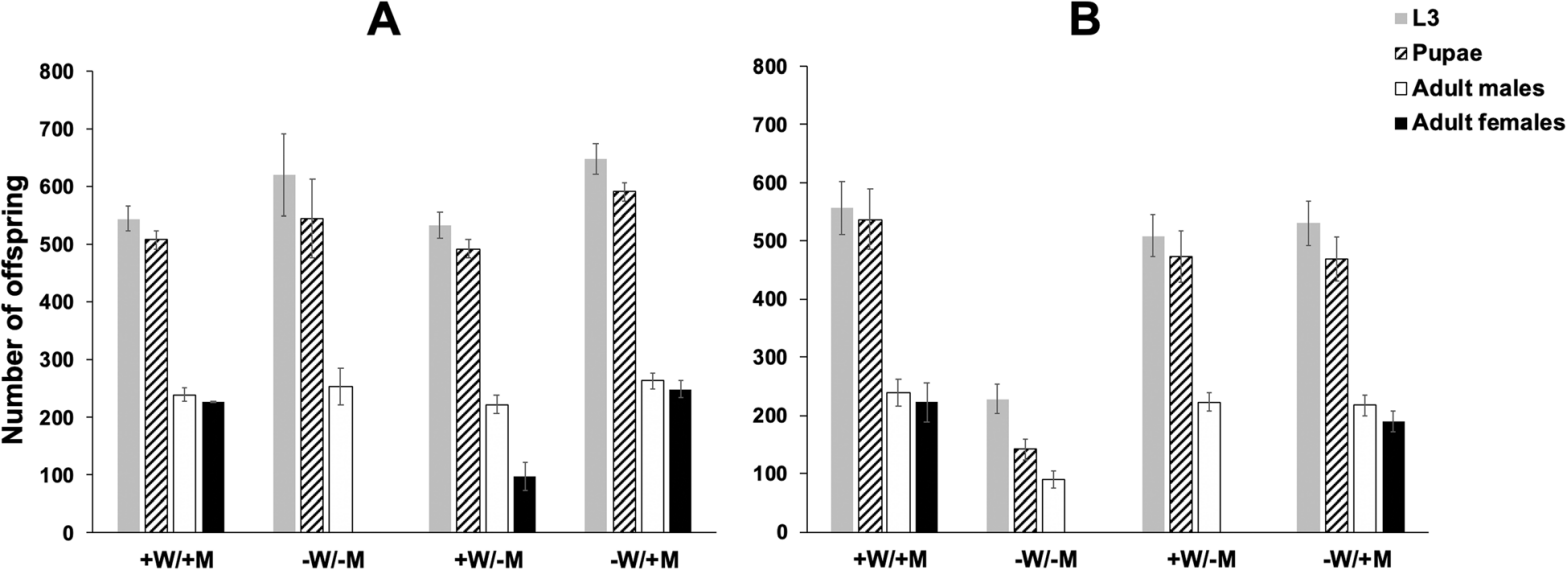
Female-specific lethality of FL3#2 (**a**) and DH6 (**b**) under different tetracycline feeding regimens. Containers were set with eight pairs of adults and the number of third instar (L3), pupae and adult male and female offspring were counted. +W: parental generation fed water with 100 μg/mL tetracycline from day 1 (D1) to D8; −W: parental generation fed water without tetracycline from D1 to D8; +M: ground meat (larval diet) with 100 μg/g tetracycline; −M: meat without tetracycline. Each experiment was performed three times. Mean ± standard deviation are shown

The rearing and female killing efficiencies of FL3#2 and DH6 were compared under different tetracycline feeding regimens. In all experiments, the larvae of the parental generation were raised on diet supplemented with a high dose of tetracycline (100 μg/mL), then 8 pairs of adults were crossed in a rearing container for each test. These adults were supplied with water that was supplemented with tetracycline (100 μg/mL) (+W) or with water that lacked tetracycline (−W). Their offspring were reared on ground meat with (+M) or without (−M) tetracycline (100 μg/g). Firstly, females of FL3#2 and DH6 were fully viable and produced similar number of offspring if the parental generation and their larval offspring were fed diet that contained high levels of tetracycline (Fig. 3a, b, +W/+M). When the parental generation and their offspring were raised on larval diet that had no tetracycline, FL3#2 produced few, if any, female adults (average 0.7 ± 0.4) while DH6 produced none (Fig. 3a, b, −W/−M). However, under such conditions the fecundity of DH6 after the first egg laying was much less than the parental wild type (WT) strain with very few eggs produced (data not shown). Consequently, the male production of DH6 (90.0 ± 15.3) on diet without tetracycline was significantly less than that from diet with tetracycline (239.7 ± 23.1) (*P* < 0.001, one-way ANOVA; Fig. 3b). In a previously described *L. cuprina* TSS (DH4), females were sterile unless fed a limiting dose of tetracycline (3 μg/mL, first 2 days after eclosion) [31]. This appeared to be due to low level expression of tTA in the ovaries activating the effector gene. We suspected a similar situation in DH6, as the tTA autoregulation system could be engaged when tetracycline was absent from the adult female diet. If so, the accumulation of tTA in the ovary would activate the *Lshid* ^*Ala2*^ effector, which could lead to female sterility.

To restore the female fertility and increase the male production of DH6, different doses of tetracycline were fed to the parental generation. We tested three different limiting tetracycline doses; 3 μg/mL for the first 2 days, 3 μg/mL for the first 8 days and 10 μg/mL for the first 8 days after emergence. Egg laying is typically at day 8. However, with each of these tetracycline feeding regimens, DH6 females were sterile (data not shown). Consequently, we supplied DH6 adults high levels of tetracycline (100 μg/mL) for the first 8 days. By doing so, fertility was fully restored and male production was 223.7 ± 14.9 (Fig. 3b, +W/−M), which is comparable to that obtained with tetracycline supplied in the adult and larval diets (+W/+M). Importantly, the high dose of tetracycline supplied to parents was not sufficient to inhibit activation of the lethal systems in DH6 as 100% of the female offspring died (Fig. 3b, +W/−M). On the other hand, FL3#2 produced 98.0 ± 24.0 females (Fig. 3a, +W/−M), which was significantly higher than that from the non-tetracycline condition (*P* = 0.001). This suggested that maternal tetracycline inhibited tTA overexpression in some FL3#2 females. FL3#2 and DH6 females were rescued by adding tetracycline to the larval diet (Fig. 3a, b, −W/+M), which indicated that females were not dying at the embryo stage or early larval stage.

**Fig. 4:**
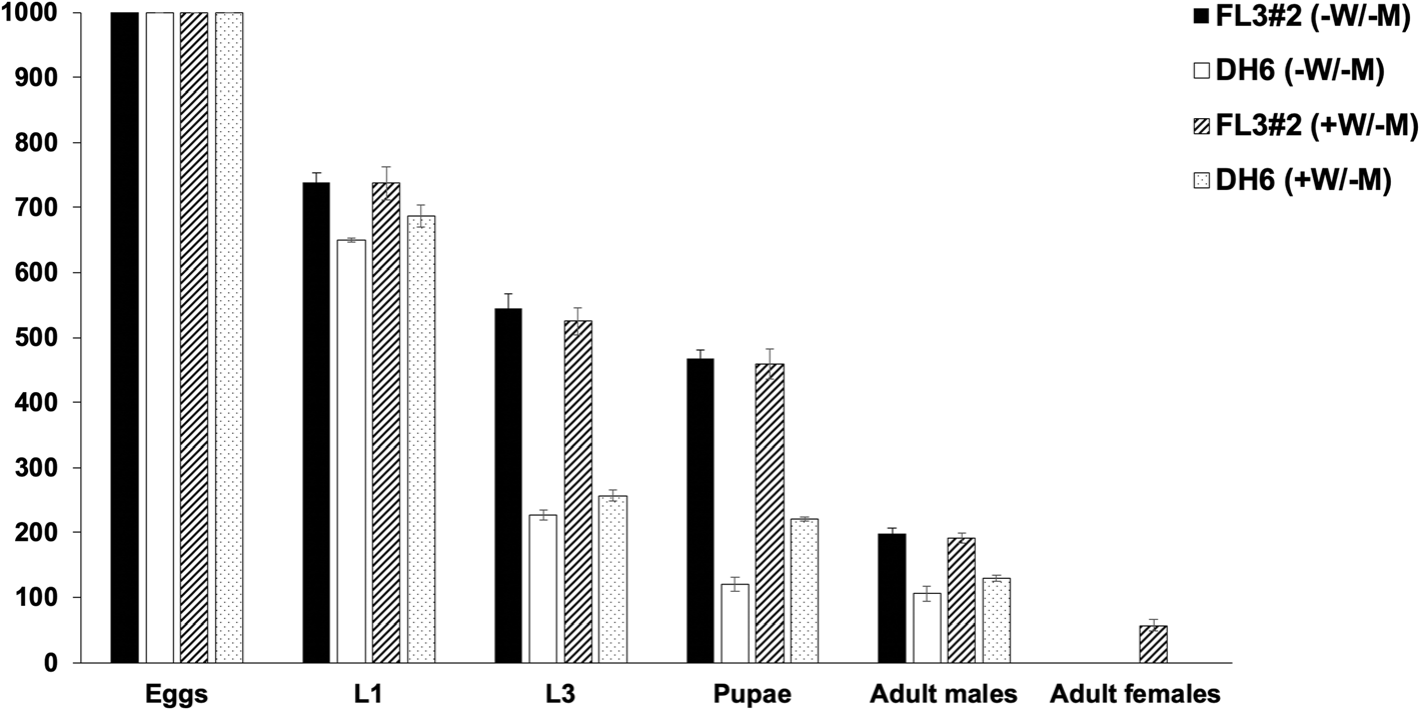
Staged lethality of FL3#2 and DH6 under different tetracycline feeding regimens. 1000 embryos were collected and the numbers of first instar (L1), third instar larvae (L3), pupae, adult males and adult females were recorded. Each experiment was performed three times. Mean ± standard deviation are shown

To further verify the effect of maternal tetracycline as well as the stage of lethality, 1000 eggs were collected from the homozygous FL3#2 and DH6 and the number of hatched first instar larvae, third instar larvae, pupae and adult males and females were counted. On diet without tetracycline, less than half of FL3#2 pupae emerged as males (42.3%), while most of DH6 pupae emerged into males (88.3%, Fig. 4). This is consistent with previous observations that FL3#2 females die at the pupal stage but indicates that DH6 females died at an earlier stage. When parents but not their offspring were fed a high level of tetracycline, FL3#2 produced 57.3 ± 8.4 female adults out of 1000 eggs while DH6 produced none (Fig. 4), which confirmed that the female lethality of DH6 cannot be inhibited by maternal tetracycline. A similar reduction from first instar to third instar in DH6 under either condition (35.0% for -W/−M, 37.4% for +W/−M) suggested that most, if not all, females survived to the third instar stage. Without tetracycline, half of DH6 third instar developed into pupae (52.8%), while most of the third instar larvae developed into pupae (86.1%) when a high level of tetracycline was supplied to the parental generation (Fig. 4). This suggested that maternal tetracycline shifted the major lethal stage from the third instar to pupae in DH6.

### Evaluation of some fitness characteristics important for mass-rearing

**Fig. 5:**
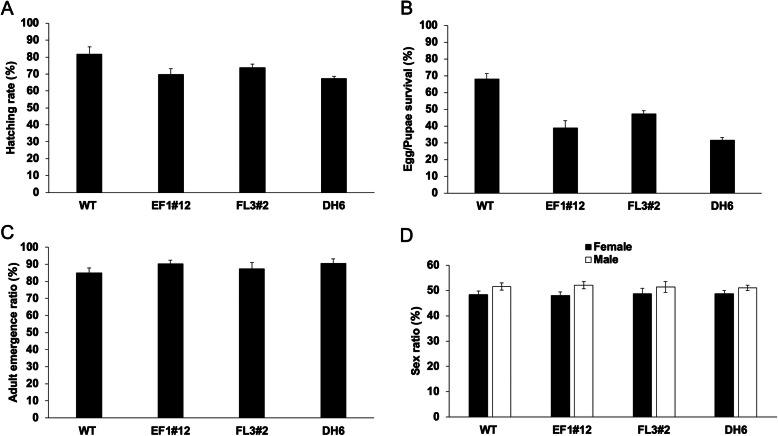
Fitness parameters of *L. cuprina* TSS. Homozygous FL3#2 and DH6 were raised in diet containing tetracycline (100 μg/mL), while WT and effector line EF1#12 were raised in diet without tetracycline. **a** percentage of first instars that hatch from embryos, **b** percentage of embryos that develop into pupae, **c** percentage of adults that emerge from pupae, and **d** sex ratio of emerged adults. Each experiment was performed three times. Mean ± standard deviation are shown

To evaluate the potential of the DH6 for mass-rearing in a factory, several fitness characteristics were measured and compared to the parental EF1#12 and FL3#2 strains and also to WT. For embryo hatching (Fig. 5a), there were no significant differences between the transgenic lines, but there were significant differences between the transgenic lines and the WT (*P* < 0.05, one-way ANOVA). The egg/pupae survival of DH6 was significantly lower than that from WT (*P* < 0.001), EF1#12 (*P* = 0.015) and FL3#2 (*P* < 0.001) (one-way ANOVA; Fig. 5b). This could indicate that basal expression of the two lethal effectors is reducing viability. The adult emergence ratio (Fig. 5c) and adult sex ratio (Fig. 5d) were not significantly different between any of the transgenic lines and the WT.

For application to the SIT, it is important that the TSS be reared efficiently with tetracycline diet in the factory, but also generate the necessary number of males for field release when raised on diet that lacks tetracycline. Consequently, we next further compared the rearing efficiency of DH6 with other TSS that have been generated in earlier studies. Specifically, the TSSs DH1, DH2, DH3, DH4, DH5 and FL3#2, one of the parental strains for DH6. These two component strains combined a driver that expressed tTA in embryos with a *tetO-hid* effector that was activated by tTA. The gene constructs are shown schematically in Additional file 1, Fig. S1. DH1 contains a *Lsbnk*-tTA embryo driver (*Lsbnk* is the *bnk* gene promoter from *L. sericata*) combined with a EF1 effector. DH2 contains the same driver but an EF3 effector (similar to EF1 but contains the wild type version of *Lshid*) [12]. DH3 contains a *Lsspt*-tTA driver (*Lsspt* is the *spitting image* gene promoter from *L. sericata*) that has activity throughout development combined with a EF3 effector, whereas DH4 contains the same driver and a EF1 effector [31]. DH5 contains *Lcact5C*-tTA driver (*Lcact5C* is the *actin5C* gene promoter from *L. cuprina*) that has activity throughout development and a EF1 effector [31]. From 24 pairs of flies on tetracycline (Table 1, Additional File 1, Fig. S1), FL3#2 produced the lowest number of adults (1314) and DH1 produced highest number of adults (2518). On diet without tetracycline or a low dose feeding regimen, DH6 produced the lowest number of males (270) and DH3 the highest (981). The number of male offspring from DH6 was 885 with a high concentration of tetracycline supplied only to the parental generation (Table 1). Thus, under such conditions the male production of DH6 is comparable to the best of the previously made TSS. The adult eclosion ratio (AER) in the release generation is also an important factor as sterile pupae are the end product from the mass rearing factory. The DH6 AER was 62.3 when tetracycline was only supplied to the parental generation. This was significantly less than the AER for other TSS such as DH1 (AER = 90; *P* < 0.001, *χ* ^2^ = 259.03) as well as WT.

## Discussion

The SIT has been successfully used to control a number of significant insect pests, including the eradication of invasive pests. For example, *C. hominivorax* was recently eradicated from the Florida Keys within a few months after detection [32]. This was achieved through successive releases of radiation sterilized males and females produced at the mass rearing facility in Panama and flown to Florida. Similarly, the SIT was used to eradicate an outbreak of *C. hominivorax* in Libya in the 1980s [33]. In addition to the SIT, eradication was achieved through the coordinated implementation of other pest control measures such as the use of insecticides to treat animals with infestations.

The DH6 TSS obtained in this study offers several advantages for an SIT program. First, a male-only release would increase the efficiency and cost-effectiveness of a population suppression program [34, 35]. Second, as female lethality cannot be inhibited by maternal tetracycline, any adult females that accidently escape from the SIT facility would not be able to produce female offspring. Third, as the lethal effect was dominant, males could be released without radiation treatment, which could potentially increase the fitness of released insects [36] and reduce the capital costs of the SIT facility [18]. Regarding a fertile male release, we [12] and others [37] previously considered that two component driver-effector systems would only be used for sterile release programs as the transgenes would independently segregate after mating. However, a recent modeling study has shown that a release of fertile males with driver and effector transgenes on different chromosomes could be effective for population suppression [38]. These three advantages are shared with other *L. cuprina* TSS (DH1 and DH5) made in earlier studies [12, 31] . One unique advantage of DH6 is that since both tTA and *Lshid* ^*Ala2*^ contain the sex-specific *Chtra* intron, only females would produce the effector proteins, which could improve male fitness compared to other TSS that use strong tTA driver lines. Another unique advantage of DH6 is the combination of two tTA-dependent lethal effectors, which would be predicted to improve strain stability under mass rearing and could reduce the risk of resistance in the field if fertile males are released. The last feature is very important in large scale long-term suppression programs [24, 30].

For a fertile release, resistance could emerge due to standing genetic variation in the targeted population [28]. For example, we recently found genetic background had a significant impact on the level of survival of female *D. melanogaster* that carried one copy of a female-specific tTA overexpression transgene [28]. Under mass rearing conditions, a TSS would be predicted to acquire random new mutations. It is possible that these mutations would provide a mechanism of resistance to the tTA overexpression system. For example, low tTA protein accumulation due to mutation in the enhancer/promoter or tTA coding sequence. The addition of the *tetO-hid* second lethal system would improve strain stability as the level of tTA protein required to activate *hid* is less than needed to cause dominant lethality based on tTA overexpression.

Despite the advantages mentioned above, DH6 does come with some limitations that could potentially hinder its practical application. First, female fertility was poor unless high levels of tetracycline were supplied in the adult diet, adding to the cost of rearing. Second, the adult eclosion ratio on diet without tetracycline was low compared to TSS made previously. This will add to the cost of the SIT program if a significant percentage of males in the release generation consume larval diet but do not develop into adults. Third, DH6 would provide little savings in larval diet costs as females die either at late-larval stage without tetracycline, or at pupae stage with maternal tetracycline. Fourth, as both DH6 lethal effectors are dependent on tTA, a complete loss-of-function mutation in the tTA gene would shut down the expression of both tTA and *hid*, thus females would be viable and fertile in the absence of tetracycline. This could be particularly problematic in a fertile release program. For this reason, it has been suggested that TSS be developed carrying two completely independent lethal systems. For example, use the quinic acid-regulated Q system to control male sterility [30], or temperature-system lethal [24], in addition to a tetracycline-repressible female lethal system.

If fertile DH6 males are released, transgenic male larvae will survive and develop in the wounds in live sheep and in dead animals. The latter is because, unlike *C. hominivorax*, *L. cuprina* is not an obligate parasite. The presence of live transgenic larvae in sheep may not be acceptable to farmers. In addition, during a suppression program we would anticipate that farmers would be particularly vigilant for flystrike and treat infested sheep with insecticides, as was done during the screwworm eradication program [16, 17]. The insecticide treatment would kill the male larvae, which would decrease the advantage of a fertile release program compared to releasing radiation sterilized males. For use in Australia, it would be desirable to backcross DH6 to a local strain of *L. cuprina* for at least 5 generations. The strain would then need to be made homozygous again for the two transgenes. Additional fitness tests for traits important for mass rearing (e.g. fecundity, egg hatch) and performance in the field (e.g. male competitiveness) would then need to be performed, as we have done previously for transgenic screwworm strains [23]. Lastly, although DH6 could be used for population suppression, each transgene could persist separately in the remaining population unless the gene has a fitness cost. There could be a negative fitness cost due to low level gene expression in females, expression of the marker gene or impact on expression of genes located near the transgene. Nevertheless, it could be more challenging to obtain regulatory approval for a field trial compared to a strain with a single dominant lethal transgene, which would not be expected to persist in the field for long after release as was observed in Brazil [39]. If so, it would be advantageous to combine the two effectors into a single construct.

Late-stage female lethality could be a beneficial for other pest species such as mosquito disease vectors that have strong density-dependent effects, since the larvae carrying lethal transgene(s) would compete for limited resources and thus reduce the survival of their wild counterparts [10, 40]. The two lethal effector approach described in this study could be applied to mosquitoes. tTA overexpression strains [10] and effector strains using the pro-apoptotic *michelob-x* gene [41] have been developed for *Aedes aegypti*. Combining these strains for the two-lethal effector would kill both sexes since the sex-specific intron is not present in these systems. The *Chtra* intron used in this study to achieve female-specific lethality would likely not be functional in mosquitoes as they appear to lack an ortholog of the *transformer* gene [42]. Alternatively, the female-specifically spliced intron from *A. aegypti Actin-4* gene could be considered, which was successfully used to regulate female-specific gene expression in this species [41]. In addition to applications in pest management, the strategy of the two-effector system can also be used when strong and conditional gene expression is needed. For example, we previously described transgenic *L. sericata* larvae that produce and secrete a human platelet derived growth factor (hPDGF) for enhanced maggot debridement therapy [43]. Combination of bi-sex tTA overexpression and the *tetO-hPDGF* transgene could potentially increase the larval secretion of hPDGF, and also reduce the clinic risks because the insects are expected to die at the pupal stage after medical use. One disadvantage of this approach is that it is possible that high levels of tTA could weaken the maggots and reduce their effectiveness for debridement.

## Conclusions

Here a stable TSS of *L. cuprina* (DH6) that carries two lethal effectors was generated. DH6 contains a tTA overexpression cassette and an additional *Lshid* ^*Ala2*^ effector cassette. The former is thought to be lethal due to “transcriptional squelching” or interference with ubiquitin-dependent protein degradation while lethality of the latter is due to widespread apoptosis. Both tTA and *Lshid* ^*Ala2*^ genes are interrupted by a sex-specific intron so only females die. The female lethality of DH6 was dominant and cannot be suppressed by maternal tetracycline. We argue that combining two different lethal effectors in a single SIT strain would increase stability during mass rearing and reduce the emergence of resistance in the field in a fertile male release program. The two lethal effector strategy could be applied to other pest species such as mosquito disease vectors and could be advantageous when high levels of conditional expression of a protein is required such as for production of wound healing factors by germ-free *L. sericata* maggots.

## Methods

### Fly rearing and double homozygous line breeding

The LA07 WT strain of *L. cuprina* was maintained as previously described [20]. In brief, adults were kept in mesh cages at 22 °C and fed a sugar/water/protein biscuit diet. Larvae were raised on 93% ground beef at 27 °C and pupae were kept in a 27 °C incubator until eclosion. Homozygous virgin females from EF1#12 were crossed with homozygous males from FL3#2 to generate double heterozygous female-specific lethal strain. The double heterozygous strain was inbred and their progeny screened to select only individuals homozygous (DH6) for both EF1 and FL3 transgenes by epifluorescence microscopy based on fluorescence intensity of ZsGreen and DsRed. Prior to testing, DH6 were maintained on diet supplemented with 100 μg/mL tetracycline for at least 5 generations with no loss of green or red fluorescence intensity, confirming the accuracy of the initial selection of homozygous larvae.

### Female lethality assessments and tetracycline feeding tests

To assess female lethality in a double heterozygous condition, 8 newly emerged males from DH6 and 8 newly emerged virgin females from WT were put in one bottle and kept on tetracycline-free adult diet for 8 days. Then embryos of 24 h egg lay intervals were reared on tetracycline-free raw ground beef (93% protein and 7% fat) and the number of third instar larvae, pupae and adult male and female were counted. Female lethality in a double homozygous condition was addressed in the same way. To test if the lethality is repressible, tetracycline (100 μg/mL) was added to water fed to the adults and to the raw ground beef fed to the larvae. To verify the lethal stage, embryos were collected on ground beef then transferred to moist black filter paper in a Petri dish and counted. Each Petri dish held 1000 embryos and was incubated at 27 °C overnight. The following day, unhatched eggs were scored and the number of first instar larvae were calculated as (1000 - number of unhatched eggs). Then the first instar larvae were transferred to meat, and the number of 3rd instar larvae, pupae, adult males and females were recorded afterwards. All lethality tests were done in triplicate.

### Fitness tests

Fitness tests were performed for the WT and transgenic lines as described previously for *C. hominivorax* [23]. Homozygous FL3#2 and DH6 were tested in diet containing tetracycline (100 μg/mL), while WT and effector line EF1#12 were tested in diet without tetracycline. All tests were replicated at least three times unless otherwise indicated. For hatching rate, 1000 eggs were collected as described above and the number of hatched larvae were scored and the percentage egg hatch was calculated. The hatched larvae were then transferred to meat and developed into pupae. The number of pupae were counted and the egg/pupae survival rate was calculated. The adult emergence ratio was calculated as [number of adults emerged/ number of pupae] X 100. Then the pupae were placed in a closed container and adults were allowed to emerge for 5 days after the emergence of the first insect. Males and females were counted and percentage of emergence and sex ratio calculated.

### Statistical analysis

Statistical analysis was performed using SigmaPlot 12.5. The differences in offspring number from different tetracycline feeding regimen for each TSS, or the differences in fitness parameters from different transgenic lines and WT, were analyzed using one-way ANOVA and means were separated using Holm-Sidak method. Differences in the adult eclosion ratio between strains were determined using a χ2 test.

## Supplementary Information

**Additional file 1: Fig. S1.** Schematic illustration of gene constructs and female lethality of *L. cuprina* transgenic sexing strains. Tetracycline feeding conditions were as follows: “-” stands for no tetracycline in the diet, “+” stands for plus tetracycline in the diet, “+/−-” indicates parents fed a low dose of tetracycline (1 or 3 μg/mL for the first two days), and “++/−” indicates a high dose of tetracycline (100 μg/mL) was supplied to the parental adults for the first eight days but not their progeny that were counted. AER stands for adult emergence ratio. The data for FL3#2 were collected up to two times from 10 to 20 pairs of adults, and all other data were from three replicates of 8-pairs per cage. A. FL3 was a tTA autoregulated construct with the female-specifically spliced intron from the *C. hominivorax* (*Chtra*) *transformer* gene. The data shown is from [20]. B. Double homozygous (DH) strain DH1 contains the driver-2 (DR2) gene cassette in which the *bottleneck* (*bnk*) cellularization gene promoter from *L. sericata* (*Lsbnk*) was used to drive expression of tTA combined with the effector-1(EF1) gene cassette in which *Lshid* ^*Ala2*^ contained the *Chtra* intron. C. DH2 contains DR2 and EF3 in which the wild type version of *Lshid* was used. The data shown for DH1 and DH2 are from [12]. D. DH3 has DR3 in which the *spitting image* (*spt*) gene promoter from *L. sericata* (*Lsspt*) was used and EF3. E. DH4 combines DR3 and EF1 lines. F. DH5 contains DR5 in which the *actin5C* gene promoter from *L. cuprina* (*Lc actin5C*) was used to drive tTA combined with EF1. The data shown for DH3, DH4 and DH5 are from [31]. G. DH6 combines FL3#2 with EF1, and data shown were from this study.

## Data Availability

All data generated or analysed during this study are included in this published article.
