# Supplementary figures and images for: Building a transgenic sexing strain for genetic control of the Australian sheep blow fly Lucilia cuprina using two lethal effectors
Source: BMC Genet. 2020 Dec 18;21(Suppl 2):141. doi: 10.1186/s12863-020-00947-y (PMC8348823; doi:10.1186/s12863-020-00947-y)

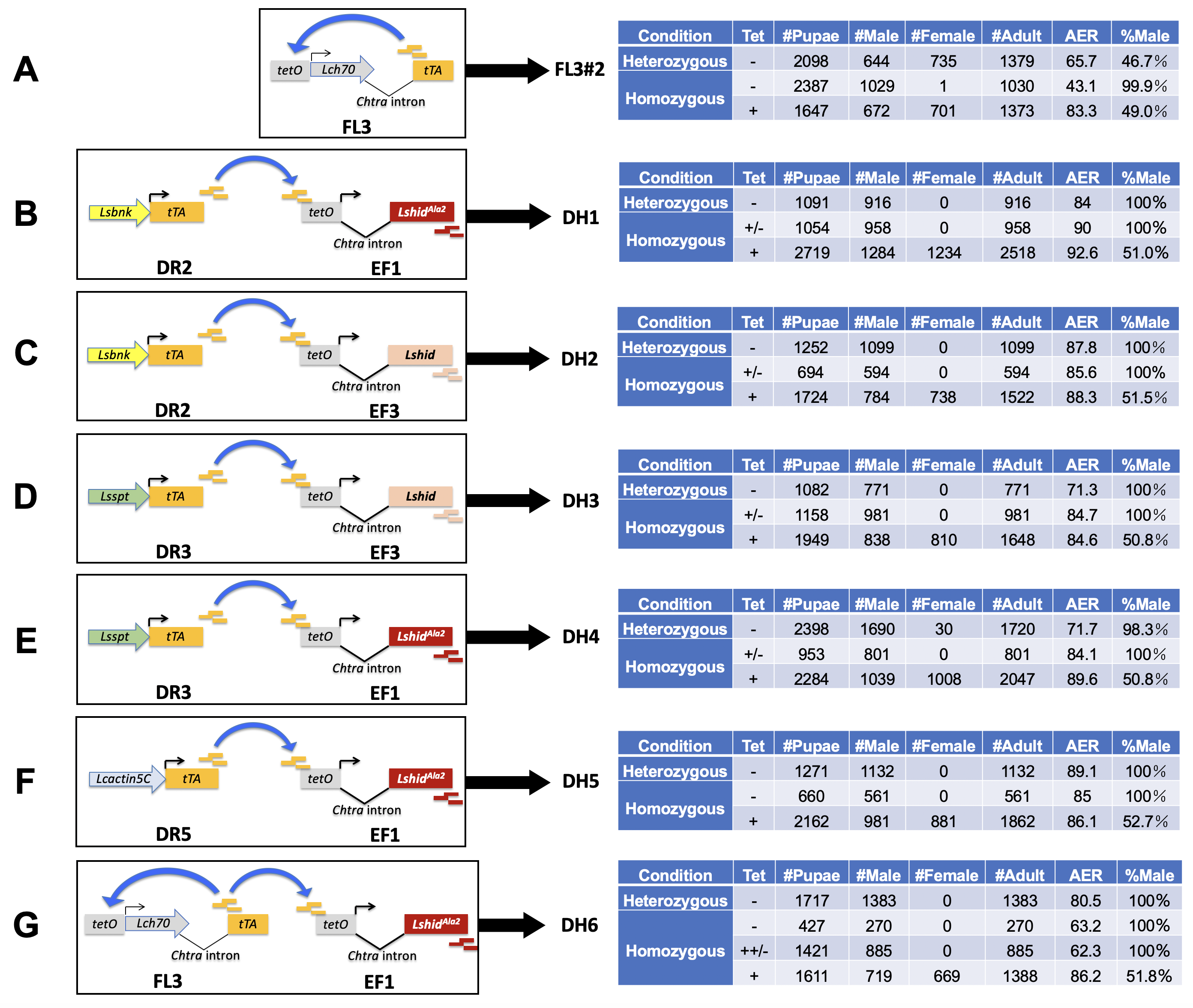

Supplement: Supplementary file 1 — Additional file 1: Fig. S1. Schematic illustration of gene constructs and female lethality of L. cuprina transgenic sexing strains. Tetracycline feeding conditions were as follows: “-” stands for no tetracycline in the diet, “+” stands for plus tetracycline in the diet, “+/−-” indicates parents fed a low dose of tetracycline (1 or 3 μg/mL for the first two days), and “++/−” indicates a high dose of tetracycline (100 μg/mL) was supplied to the parental adults for the first eight days but not their progeny that were counted. AER stands for adult emergence ratio. The data for FL3#2 were collected up to two times from 10 to 20 pairs of adults, and all other data were from three replicates of 8-pairs per cage. A. FL3 was a tTA autoregulated construct with the female-specifically spliced intron from the C. hominivorax (Chtra) transformer gene. The data shown is from [20]. B. Double homozygous (DH) strain DH1 contains the driver-2 (DR2) gene cassette in which the bottleneck (bnk) cellularization gene promoter from L. sericata (Lsbnk) was used to drive expression of tTA combined with the effector-1(EF1) gene cassette in which Lshid Ala2 contained the Chtra intron. C. DH2 contains DR2 and EF3 in which the wild type version of Lshid was used. The data shown for DH1 and DH2 are from [12]. D. DH3 has DR3 in which the spitting image (spt) gene promoter from L. sericata (Lsspt) was used and EF3. E. DH4 combines DR3 and EF1 lines. F. DH5 contains DR5 in which the actin5C gene promoter from L. cuprina (Lc actin5C) was used to drive tTA combined with EF1. The data shown for DH3, DH4 and DH5 are from [31]. G. DH6 combines FL3#2 with EF1, and data shown were from this study. [file 12863_2020_947_MOESM1_ESM.png]
